# Herpesvirus-Associated Central Nervous System Diseases after Allogeneic Hematopoietic Stem Cell Transplantation

**DOI:** 10.1371/journal.pone.0077805

**Published:** 2013-10-04

**Authors:** Meiqing Wu, Fen Huang, Xinmiao Jiang, Zhiping Fan, Hongsheng Zhou, Can Liu, Qianli Jiang, Yu Zhang, Ke Zhao, Li Xuan, Xiao Zhai, Fuhua Zhang, Changxin Yin, Jing Sun, Ru Feng, Qifa Liu

**Affiliations:** Department of Hematology, Nanfang Hospital, Southern Medical University, Guangzhou, China; University of Nebraska - Lincoln, United States of America

## Abstract

Herpesvirus infections of the central nervous system (CNS) are associated with encephalitis/myelitis and lymphoproliferative diseases in immunocompromised individuals. As of now, data of herpesvirus-associated CNS diseases in transplant recipients is limited. Hence, in this prospective study, we investigated the incidence of herpesvirus-associated CNS diseases and explored the diagnosis of these diseases in 281 allogeneic hematopoietic stem cell transplantation (allo-HSCT) recipients. Herpesvirus-DNA and cerebrospinal fluid (CSF) cells were sampled from 58 recipients with herpesvirus-associated diseases or with unexplainable CNS manifestations. Results showed that 23 patients were diagnosed as herpesvirus-associated CNS diseases, including 15 Epstein-Barr virus (EBV)-associated diseases (4 encephalitis and 11 lymphoproliferative diseases), 5 herpes simplex virus type 1 encephalitis, 2 cytomegalovirus encephalitis/myelitis and 1 varicella zoster virus encephalitis. The median time of diseases onset was 65 (range 22-542) days post-transplantation. The 3-year cumulative incidence of herpesvirus-associated encephalitis/myelitis and post-transplant lymphoproliferative disorder (PTLD) was 6.3% ±1.9% and 4.1% ±1.2%, respectively. Of the evaluable cases, CSF cells mainly consisted of CD19^+^CD20^+^ B cells (7/11) and had clonal rearrangement of immunoglobulin genes (3/11) in patients with CNS-PTLD. On the contrary, in patients with encephalitis/myelitis, CSF cells were comprised of different cell populations and none of the gene rearrangement was detected. Herpesvirus-associated CNS diseases are common in the early stages of allo-HSCT, wherein EBV is the most frequent causative virus. The immunophenotypic and clonal analysis of CSF cells might be helpful in the differential diagnosis between encephalitis and lymphoproliferative diseases.

## Introduction

Herpesviruses, the family of neurotropic viruses, may cause encephalitis/myelitis of various degrees of severity in immunocompetent individuals [1,2]. Epidemiological studies demonstrate that α-herpesviruses, such as herpes simplex virus type 1 (HSV-1) and varicella zoster virus (VZV), are the most frequent etiological agents found in sporadic viral encephalitis/myelitis [2,3]. β- and γ-herpesviruses, such as cytomegalovirus (CMV), Epstein-Barr virus (EBV) and human herpes virus 6-8 (HHV6-8), are known to cause encephalitis/myelitis, but it is rare in immunocompetent individuals [3,4]. Recently, a growing body of data suggests that encephalitis/myelitis, even lymphoproliferative diseases, resulting from β- and γ-herpesviruses are not rare in immunocompromised individuals including transplant recipients [5-9]. However, these were mainly limited to case reports and retrospective analysis [8,10,11]. To date, there is an absence of large prospective studies about herpesvirus-associated central nervous system (CNS) diseases in recipients of allogeneic hematopoietic stem cell transplantation (allo-HSCT).

In immunocompromised individuals, herpesvirus-associated CNS diseases, such as encephalitis/myelitis and lymphoproliferative diseases, are representative of acute complications [12-14]. Since specific therapy is limited to only several viral agents, accurate diagnosis and early therapy reduces the extent of permanent injury in survivors and positively influences survival rate [15]. Diagnosis of herpesvirus encephalitis/myelitis mainly depends on the neurological manifestations, virus in cerebrospinal fluid (CSF) as well as neuroimaging [16], whereas diagnosis of lymphoproliferative diseases requires CNS biopsy [17,18]. Currently, polymerase chain reaction (PCR) testing of virus-DNA in CSF is a high sensitive and specific method to diagnose herpesvirus-associated CNS diseases [19]. In recipients of allo-HSCT, most post-transplant lymphoproliferative disorder (PTLD) occurs in the early stages of transplantation, and the platelet counts of some patients are too low to perform CNS biopsy. Thus, in clinical practice, the diagnosis of CNS-PTLD is dependent on the clinical manifestations, detection of the virus in CSF, cytomorphology of CSF cells and neuroimaging or autopsy [14,19,20]. In this prospective study, we investigated the incidence of herpesvirus-associated CNS diseases and explored the diagnosis of these diseases in the recipients of allo-HSCT.

## Patients and Methods

### Patients

Patients undergoing allo-HSCT were eligible for the study if they fulfilled the following criteria: (1) patients with EBV-associated diseases; (2) patients with other herpesvirus-associated diseases accompanying CNS manifestations; (3) patients with unexplainable CNS manifestations. According to the criteria, 58 of 281 patients undergoing allo-HSCT between July 2008 and September 2012 were enrolled in this study: 39 with EBV-associated diseases, 11 with other herpesvirus-associated diseases, and 8 with unexplainable CNS manifestations. Moreover, 17 patients with herpesvirus-DNA-emia (EBV in 9 and CMV in 8) who did not develop herpesvirus-associated diseases and 10 patients who were negative for herpesvirus-DNA volunteered to have their CSF monitored as controls (platelet >50×10^9^/L). Of the 85 enrolled patients, 39 were females and 46 males, and the median age was 28(range 14-53) years. The primary diseases included leukemia (n=74), aplastic anemia (n=5), lymphoma (n=4), and myelodysplastic syndrome (n=2). This study was performed in accordance with the modified Helsinki Declaration, and the protocol was approved by the Ethics Committee of Southern Medical University affiliated Nanfang Hospital before study initiation. All donors, recipients and/or guardians provided written informed consent prior to study enrollment.

### Transplantation

Forty-six patients received related donor and 39 received unrelated donor transplants. Forty-seven received HLA-matched and 38 HLA-mismatched grafts. The conditioning regimens and graft versus host disease (GVHD) prophylaxis were based on our previous description [21].

### Infection prophylaxis

Infection prophylaxis was administrated according to the literature [22,23]. Oral sulfamethoxazole was given to all patients. Acyclovir was given daily from the conditioning to engraftment, and then it was administered daily for 7 days every 2 weeks until 1 year post-transplants for virus infection prophylaxis. Ganciclovir was given for 2 weeks before transplantation for prophylaxis of CMV infection, and then again during periods of CMV viremia within 1 year after transplantation. Antifungal agents were administered 5 days pre-transplants and continued for +30 to +90 days post-transplantation according to the history of invasive fungal infection.

### Herpesvirus-DNA monitoring in blood and CSF

The real-time quantitative PCR was used to detect EBV- and CMV-DNA, and qualitative PCR was used for other herpesviruses. According to the manufacturer (ZJ Bio-Tech Co., Ltd., Shanghai, China), it was defined as positive if EBV- or CMV-DNA were higher or equal to 500 copies/ml in plasma or CSF.

The blood EBV- and CMV-DNA were monitored in recipients weekly for the first 3 months, every 2 weeks between 4 to 9 months, monthly between 10 to 24 months and every 3 months between 25 to 36 months post-transplantation. If virus-DNA was positive, it would be monitored twice a week. Herpesviruses other than EBV and CMV were not routinely monitored, and these were only performed on the suspicious patients. For the patients enrolled in this study, paired blood and CSF samples were detected for herpesvirus-DNA during disease and follow-up. Meanwhile, other pathogens such as adenovirus, human parvovirus B19, BK virus, as well as bacteria and fungus were monitored for their presence in blood and CSF.

### Other parameters monitoring

Once herpesvirus-associated CNS diseases were suspected, other parameters, including CSF cells and magnetic resonance imaging (MRI) were performed within 7 days after the disease onset. The immunophenotype of CSF cells were assayed by FACSCantoTM II (BD Biosciences) and the acquired data were further analyzed using BD-FACSDiva Software. The rearrangement of immunoglobulin gene and T-cell receptor in CSF cells was detected by PCR as described previously [24,25].

### Diagnosis

Herpesvirus-associated CNS diseases include encephalitis/myelitis and PTLD. The diagnosis of herpesvirus encephalitis/myelitis was according to the criteria of the International Herpes Management Forum, including: (1) neurological signs or symptoms; (2) a positive virus-DNA in CSF; (3) inflammatory changes or normal images in neuroimaging; (4) absence of other etiologic evidence or established diseases [16]. The diagnosis of herpesvirus-associated CNS-PTLD was based on the criteria of World Health Organization and European Conference on Infections in Leukemia as well as the literature [18,26]. Definitive diagnosis of PTLD required histopathological evidence and virus gene or productions identified in the lesions. For the patients with unavailable biopsy, CNS-PTLD was defined as following: (1) CNS clinical manifestations or abnormal neuroimaging; (2) virus-DNA positive in CSF; (3) CSF cells revealing monoclonality by flow cytometry or PCR, or cytomorphological proof of atypical lymphocyte; (4) absence of other etiologic evidence or established diseases.

### Statistical analyses

The SPSS software package was used for data analysis. Comparisons of categorical variables were made by means of chi-squared test. Differences between numerical variables were calculated by the Mann-Whitney U-test. The incidence of diseases was estimated by the method of Kaplan-Meier and comparisons were made with Log-rank test. All P values were 2-sided with significance level at α=0.05.

## Results

### Herpesvirus serology and virus-DNA of recipients and donors before transplantation

Of the patients enrolled in this study, the seroprevalence of HSV-1, HSV-2, VZV, EBV, CMV, HHV-6, HHV-7 and HHV-8 was 28.2%, 15.3%, 37.6%, 84.7%, 90.6%, 42.4%, 25.9% and 8.2% in recipients, and it was 23.5%, 9.4%, 41.2%, 89.4%, 92.9%, 47.1%, 29.4% and 5.9% in donors, respectively, before transplantation. Four donors were CMV-DNA positive and one was EBV-DNA positive in blood during health examination, and they became virus-DNA negative at the time of stem cell collection after antiviral treatment. Other herpesvirus-DNA of blood was negative in both recipients and donors at the time of transplantation.

### Aetiology in CSF and herpesvirus-associated CNS diseases

Of the 85 enrolled patients, 23 patients were CSF herpesvirus-DNA positive, including EBV (n=14), HSV-1 (n=5), CMV (n=2), VZV (n=1), and EBV/CMV co-infection (n=1). These positive patients were 14/39 EBV-associated diseases, 8/11 other herpesvirus-associated diseases, and 1/8 patients with unexplainable CNS manifestations, while 27 control patients were all herpesvirus-DNA negative in CSF. The viruses detected in the CSF were consistent with the viruses found in blood in all patients except for one patient, who was CSF EBV-DNA positive and blood EBV-DNA negative. The serostatus prior to transplantation for the patients who developed CNS herpesvirus diseases are showed in Table 1.

**Table 1:** The serostatus prior to transplantation for the patients (n=23) who developed CNS herpesvirus diseases by the respective virus.

Virus	Serostatus before transplantation		Patients(n)
	Donor	Recipient	
EBV	+	+	10
	+	-	3
	-	-	2
HSV-1	+	+	3
	+	-	1
	-	+	1
VZV	+	+	1
CMV	+	+	2

Note: + positive; - negative.

**Figure 1:**
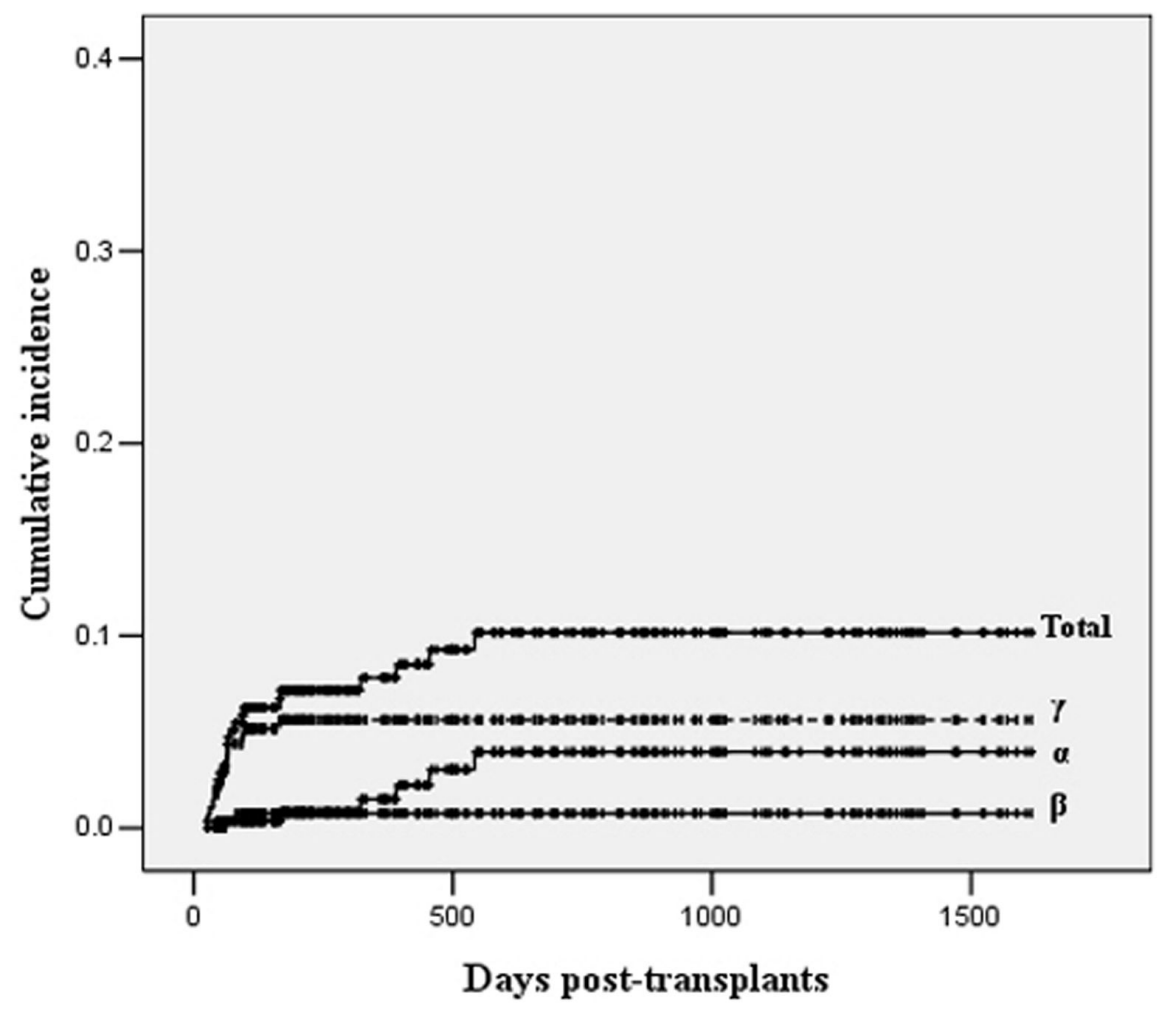
The cumulative incidence of herpesvirus-associated CNS diseases.

According to clinical manifestations and virus-DNA of the CSF as well as other parameters, 23 herpesvirus-associated CNS diseases were diagnosed, including 4 EBV encephalitis, 5 HSV-1 encephalitis, 2 CMV encephalitis/myelitis, 1 VZV encephalitis, and 11 EBV-associated CNS-PTLD. Of the 11 patients with EBV-associated CNS-PTLD, 8 cases were systemic PTLD accompanying with CNS involvement and 3 were isolated CNS-PTLD. The median time of disease onset was 65 (range 22-542) days post-transplantation and 73.9% (17/23) patients occurred within 100 days. The 3-year cumulative incidence of total, α-, β- and γ- herpesvirus-associated CNS diseases was 10.2% ± 2.1%, 4.0% ± 1.7%, 0.8% ± 0.5% and 5.6% ± 1.4%, respectively (Figure 1). The incidence of herpesvirus-associated encephalitis/myelitis and PTLD was 6.3% ±1.9% and 4.1% ±1.2%, respectively. EBV-associated CNS diseases were the most frequent episode (5.6% ± 1.4%), followed by HSV-1 (3.4% ±2.0%), CMV (0.8% ± 0.5%) and VZV (0.7% ± 0.7%).

### Other parameters in CSF

Of the 23 patients with herpesvirus-associated CNS diseases, 15 cases had elevated CSF pressures (median 1.96 kpa, range 0.78-2.74 kpa), 13 had high CSF protein(median 0.77g/L, range 0.20-1.85 g/L) and 17 patients were pleocytosis (median 64/µl, range 0-400/µl). Atypical lymphocyte was found in 8 patients who were diagnosed as CNS-PTLD. The immunophenotypic analysis of CSF cells was available in 17 patients and gene rearrangement was observed in 5 patients. Results showed that CSF cells mainly consisted of CD19^+^CD20^+^ B cells(n=7) and different cell populations of CD3^+^ T cells, CD19^+^ B cells and CD14^+^ monocytes(n=2) in patients with CNS-PTLD. In the patients with encephalitis/myelitis, CSF cells were comprised of different cell populations (n=8). The gene rearrangement of CSF cells revealed clonal rearrangement of immunoglobulin in 3 patients with PTLD, and none of the gene rearrangement was detected in 2 patients with encephalitis. The cell populations presented in CSF were identical to those infiltrated in the available extracerebral biopsy. Moreover, the CSF of the 23 patients with herpesvirus-associated CNS diseases was cultured negative for bacteria and fungi.

### The imaging characteristics

**Figure 2:**
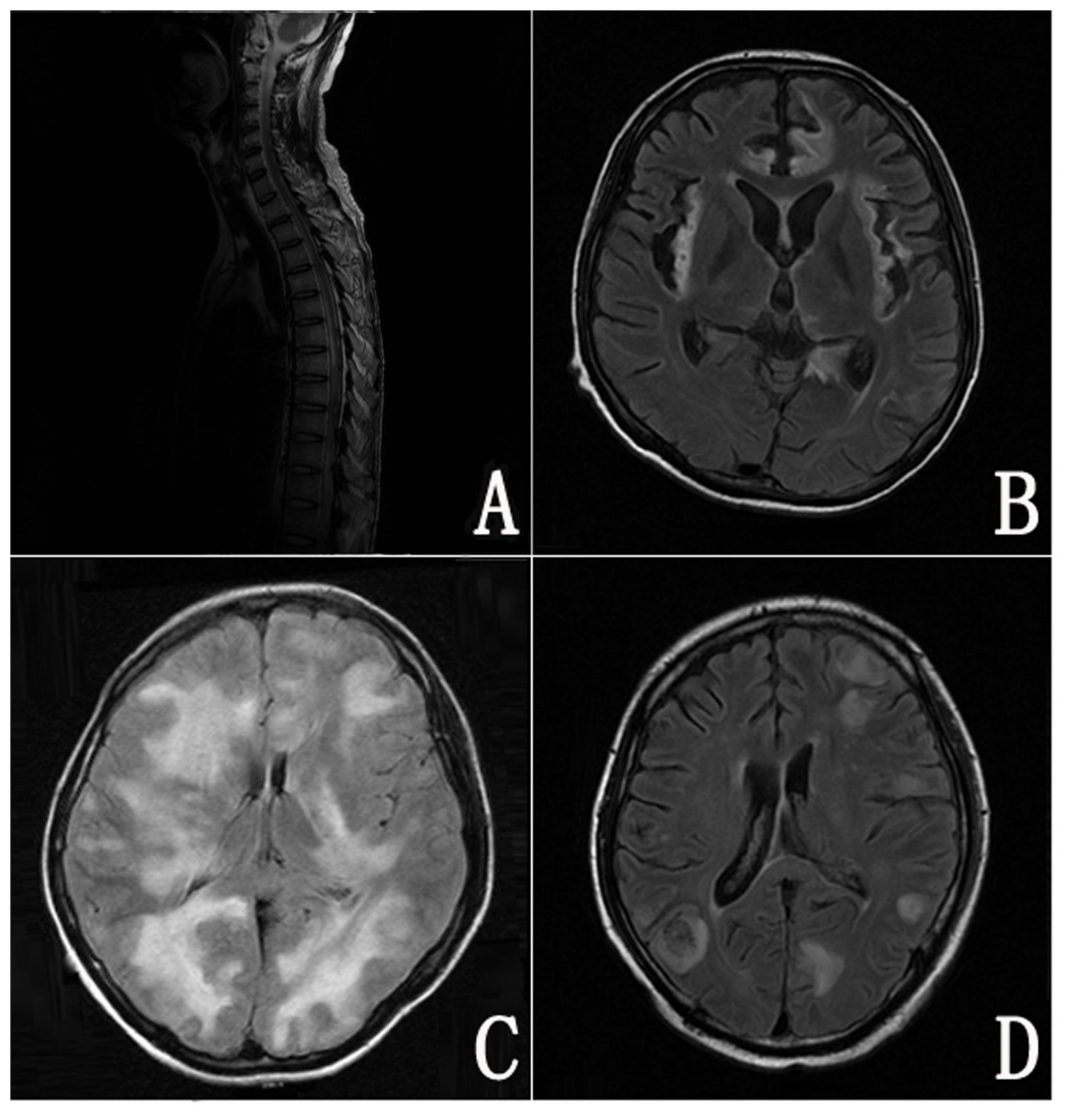
MRI of patients with herpesvirus-associated CNS diseases. A: T2-weighted imaging of a patient with EBV and CMV co-infection reveals disseminate abnormality signals changes in spinal cord; B: MRI of a patient with HSV-1 encephalitis shows bilateral and symmetric distribution of hyperintense lesions involving the frontal and temporal lobes, insula and splenium of corpus callosum on FLAIR image; C: Large patchy areas of increased signals are seen in bilateral white matter under the cortex on FLAIR image in a patients with EBV encephalitis; D: Multiple cloudlike lesions adjacent to pallium and paraventricular lesions are hyperintense on FLAIR image in a patient with EBV-associated CNS-PTLD.

MRI of the CNS at disease onset was normal in 6 and abnormal in 17 cases. Abnormality included focal or diffuse inflammatory changes in 9 patients and space-occupying lesions in 8 patients (Figure 2). In addition, 2 patients presented as disseminated inflammatory changes and 1 patient exhibited normal MRI at disease onset. With the development of diseases, these 3 patients had space-occupying lesions as seen in CNS imaging.

### Herpesvirus and PTLD

To identify correlations between EBV/CMV and PTLD, the status of EBV- and CMV-emia prior to PTLD and at the time of PTLD onset was analysed in the 23 patients diagnosed with PTLD (11 CNS-PTLD and 12 had extracerebral PTLD without concomitant CNS involvement). Nineteen and 10 patients had the history of EBV- and CMV-emia, respectively, before PTLD. Twenty-two and 3 patients had blood EBV- and CMV-DNA positive, respectively, at the time of disease onset. EBV-emia was directly correlated with PTLD (P<0.001), while there was no connection between CMV-emia and PTLD (P=0.310). Then, we further analysed the relation between CMV-emia and EBV-emia. Results revealed that 7/22 (31.8%) patients with EBV-emia had the history of CMV-emia within 4 weeks prior to EBV reactivation. On the contrary, 2/13 (15.4%) patients with CMV-emia had the history of EBV-emia. EBV-emia was closely related to the pre-existing CMV infection (P=0.002).

### Treatment and outcome

Twenty-two patients received therapy and one patient with PTLD abandoned treatment because of the patient’s desire and financial constraint. Immunosuppressive agents were reduced for all herpesvirus-associated CNS diseases if the patient’s condition permitted. Acyclovir was given to patients with HSV or VZV encephalitis. Ganciclovir and/or foscarnet were used for CMV. For EBV-associated CNS diseases, the treatments including antiviral agents, rituximab, chemotherapy, donor lymphocyte infusion and EBV-specific cytotoxic lymphocyte were administered. After treatments, 11 patients were complete response (1HSV-1 encephalitis, 1 VZV encephalitis, 2 EBV encephalitis and 7 PTLD), 4 partial response (1 HSV-1 encephalitis, 1 CMV encephalitis and 2 EBV encephalitis) and 8 inefficiency (3 HSV-1 encephalitis, 1 CMV myelitis and 4 PTLD). With a median follow-up of 238 (range 4-1144) days after the diagnosis of herpesvirus-associated CNS diseases, 10 patients survived (4 PTLD, 2 EBV encephalitis, 2 HSV-1 encephalitis, 1 CMV encephalitis and 1 VZV encephalitis, respectively) and 13 died. The causes of death were related with herpesvirus in 9 cases and not related with herpesvirus in 4 cases (2 acute GVHD, 1 chronic GVHD and 1 cerebral hemorrhage).

## Discussion

Herpesviruses are recognized as the most common causes of aseptic encephalitis/myelitis in immunocompetent individuals [1]. Recently, a retrospective study of large samples size from M. Schmidt-Hieber and colleagues showed that the incidence of viral encephalitis after allo-HSCT was 1.2%, and it was mainly caused by HHV-6, followed by EBV, HSV, JC virus, CMV, VZV and adenovirus [11]. This spectrum of causative viruses associated with encephalitis differs markedly from that seen in immunocompetent individuals (mainly HSV and VZV) [27,28]. However, the investigators did not analyze the herpesvirus-associated CNS-PTLD in this retrospective study. To our knowledge, our study is the first prospective report of the largest samples about the herpesvirus-associated CNS diseases, including encephalitis/myelitis and PTLD, in the recipients of allo-HSCT. Our data showed that the 3-year cumulative incidence of herpesvirus-associated encephalitis/myelitis and PTLD was 6.3% ±1.9% and 4.1% ±1.2%, respectively. The incidence of herpesvirus encephalitis/myelitis in our study was higher as compared to the retrospective investigation (6.3% *vs.* 1.2%) [11]. The spectrum of causative viruses was also different. In our cohort, EBV was the most frequent agent, followed by HSV-1, CMV and VZV. Furthermore, the median onset time of herpesvirus-associated CNS diseases in this study was earlier than that in the study of M. Schmidt-Hieber(65 days *vs.* 106 days) [11]. The distinct study designs between our research and M. Schmidt-Hieber’s report might result in these differences. Another reason might be that EBV-associated CNS diseases were sufficiently considered in this study. EBV-DNA monitoring of CSF was routinely performed on all patients with EBV-associated diseases, whereas other herpesviruses were only detected in the suspicious patients presenting with CNS manifestations. In addition, in our cohort, the high proportion of recipients receiving anti-thymocyte globulin treatment, undergoing HLA mismatch and unrelated donor transplant, which are risk factors for EBV-associated diseases, might be additional contributing factors to the high incidence of EBV-associated CNS disease.

In transplant recipients, most of the CNS malignant diseases caused by herpesvirus manifest as lymphoproliferative diseases, which mainly results from EBV infection [17,29]. Recent data implicated that other herpesviruses, such as CMV or HHV-6, might contribute to the development of PTLD [30,31]. Tsao, et al. [30] and Brion, et al. [31] reported PTLD in the recipients of allo-HSCT with histological evidence or genome evidence of CMV and EBV co-infection in tumor tissue. Tsao, et al. presumed that CMV infection could inhibit T cell responses, which might contribute to insufficient EBV-specific T cell responses and the subsequent proliferation of EBV transformed B cells [30]. In this study, though CMV-emia was not directly correlated to PTLD at disease onset, 31.8% patients with EBV-emia had the history of CMV-emia within 4 weeks prior to EBV reactivation. Thus, our findings are in agreement with the above presumption.

According to the International Herpes Management Forum [16], the diagnosis of herpesvirus-associated encephalitis/myelitis is based on clinical manifestations virus detection in the CSF, and neuroimaging, while the definitive diagnosis of malignant diseases should be verified by histopathology [29]. As aforementioned, most PTLD occurs in the early stages post-transplantation in the recipients of allo-HSCT. These patients generally have low platelet counts thus making them unsuitable for CNS biopsy. Therefore, their diagnosis of CNS-PTLD is dependent on CNS signs and symptoms, virus detection and morphological analysis of CSF cells as well as neuroimaging [14,19,20]. Likewise, the diagnosis of CNS lymphoma also based on this diagnostic method due to the subsequent serious lesion of CNS biopsy [32]. In this study, in addition to the diagnostic method aforementioned, we introduced the analysis of immunophenotype and gene rearrangement of CSF cells to diagnose CNS-PTLD. Our results showed that in patients with CNS-PTLD, the cells of CSF mainly consisted of CD19^+^CD20^+^ B cell and had clonal rearrangement of immunoglobulin genes, and the cell populations presented in CSF were identical to those infiltrated in the available extracerebral biopsy. Whereas in patients with encephalitis/myelitis, the CSF cells were comprised of different cell populations and none of the gene rearrangements was detected. Therefore, based on these, we suggested that immunophenotypic and clonal analysis of CSF cells could be proposed as an alternative definite diagnostic method for CNS-PTLD. Interestingly, we observed 2 patients presenting as disseminated inflammatory changes and 1 patient with normal MRI had space-occupying lesions in CNS with the development of diseases. Thus, whether EBV encephalitis is an independent disease or an early manifestation of PTLD in recipients of allo-HSCT, is worth further studying.

The main drawback of this study was that we did not perform biopsy or autopsy of the CNS. The reasons for this were mainly ethical issues and that the majority of Chinese did not accept autopsy after death. In addition, we did not perform serial monitoring of herpesviruses other than EBV and CMV, which might have an impact on the incidence of the herpesvirus-associated CNS diseases.

In conclusion, herpesvirus-associated CNS diseases are common in the early stages of allo-HSCT, wherein EBV is the most frequent causative virus. The immunophenotypic and clonal analysis of CSF cells might be helpful in the differential diagnosis between encephalitis and lymphoproliferative diseases.
